# Supplementary material for: How Does the Sleep Regularity Questionnaire Relate to Wearable- and Diary-Derived Sleep Regularity?
Source: J Circadian Rhythms. 2026 May 12;24:2. doi: 10.5334/jcr.265 (PMC13178601; doi:10.5334/jcr.265)
Supplement: Supplementary file 1. — Validity of Ultrahuman Rings and mean data for Part 1 and 2 of the study. [file jcr-24-265-s1.pdf]

Supplementary Files

Validity of Ultrahuman Ring Device

Sleep was measured concurrently using the Somfit (criterion device) and Ultrahuman Ring Air (test device) across 20 nights in 4 participants (2 male, 2 female; mean ± SD age = 29 ± 6 years) to ascertain key sleep metrics used in the current study (sleep onset, sleep offset, and total sleep time).

- Somfit: Criterion data were acquired using the Somfit system, which records single-channel EEG (Fp1–Fp2), electro-oculography (Fp1–Fpz, Fp2–Fpz), electromyography (Fp1–Fp2), pulse oximetry, head position, snoring sound, movement, and ambient light. Somfit has been validated against full polysomnography (27, 28).
- Ultrahuman Ring Air: A commercial wearable ring integrating photoplethysmography (PPG), accelerometry, and skin temperature sensors. Proprietary algorithms are used to derive sleep timing and duration.

Sleep onset, sleep offset, and total sleep time were extracted for each night. Agreement between devices was assessed using Pearson correlations, intraclass correlation coefficients (ICC; two-way mixed, absolute agreement), mean difference (bias), typical error (TE), and coefficient of variation (CV%).

Results

Across 20 paired nights, agreement between devices was excellent for sleep onset (r = 0.9998, ICC = 0.9997) and sleep offset (r = 0.985, ICC = 0.974), and good for total sleep time (r = 0.889, ICC = 0.844). Average differences between devices were small (–1.4 to +3.6 min), with typical errors of 8–15 min.

Supplementary Table 1. Validity statistics (20 nights) between the Ultrahuman Ring and Somfit (criterion).

Measure	Somfit Mean ± SD	Ultrahuman Mean ± SD	Pearson's r	ICC	Mean Diff (min)	TE (min)	CV (%)
Sleep Onset	22:55 ± 1:12 h	22:55 ± 1:12 h	0.9998	0.9997	-1.4	8.3	0.9
Sleep Offset	07:17 ± 0:59 h	07:21 ± 1:02 h	0.985	0.974	+3.6	8.0	1.8
Total Sleep	7:44 ± 0:45 h	7:45 ± 0:44 h	0.889	0.844	+1.0	14.9	3.2

Supplementary Table 2. Mean data from Part 1 of the study, including objective sleep metrics collected by the smart ring over 21 days (n=31) and SRQ scores.

Metric	Mean ± SD	Range	Units / notes
Mean bedtime	21:57 ± 4.1 h	00:02 – 23:58	Clock time; within-participant mean bedtime (SD reflects between-participant dispersion of mean bedtimes)
Mean wake time	06:57 ± 0.9 h	04:28 – 08:32	Clock time; within-participant mean wake time
Sleep Regularity Index (SRI)	80.64 ± 5.61	66.78 – 90.48	−100 to +100; higher values indicate more regular timing
Interdaily stability (IS)	0.76 ± 0.07	0.53 – 0.91	Dimensionless; higher values indicate more stable, clock-time–anchored rhythms
Bedtime variability (SD of bedtime)	59.0 ± 25.7 min	25.2 – 135.7	Minutes; within-participant SD of nightly bedtime
Waketime variability (SD of waketime)	58.1 ± 24.4 min	20.6 – 125.8	Minutes; within-participant SD of nightly waketime
Social jetlag (SJL)	0.90 ± 0.88 h	0.09 – 3.92	Hours; absolute difference in midsleep between workdays and free days
Composite phase deviation (CPD)	1.10 ± 0.50 h	0.47 – 2.89	Hours; combined mistiming and night-to-night variability of midsleep
SRQ Circadian Regularity subscale	10.29 ± 2.90	2 – 15	Sum of 4 items (0–16); higher scores indicate more regular bed and wake timing
SRQ Sleep Continuity Regularity subscale	4.84 ± 2.21	1 – 8	Sum of 2 items (0–8); higher scores indicate more regular nocturnal awakenings and time awake
SRQ Global score	15.13 ± 4.11	4 – 23	Sum of 6 items (0–24); higher scores indicate more regular perceived sleep timing and continuity overall

Supplementary Table 3. Mean data from Part 2 of the study, including subjective sleep diary metrics collected over one week (n=52) and SRQ scores.

Metric	Mean ± SD	Range	Units / notes
Mean bedtime (diary-derived)	23:44 ± 1.37 h	00:06 – 23:57	Clock time; within-participant mean bedtime
Mean wake time (diary-derived)	07:43 ± 1.97 h	03:40 – 20:49	Clock time; within-participant mean wake time
Perceived sleep quality	6.69 ± 1.11	4.29 – 9.29	Diary-rated sleep quality (0–10; higher = better)
Mean total sleep time (TST)	7.53 ± 1.18 h	4.42 – 10.00	Hours; within-participant mean nightly TST
Bedtime variability (SD of bedtime)	114.9 ± 138.8 min	10 – 676	Minutes; within-participant SD of nightly bedtime
Waketime variability (SD of waketime)	87.6 ± 99.0 min	22 – 669	Minutes; within-participant SD of nightly waketime
Mid-sleep variability (SD of midsleep)	104.5 ± 150.9 min	9 – 725	Minutes; within-participant SD of nightly midsleep
TST variability (SD of TST)	75.6 ± 43.2 min	20 – 262	Minutes; within-participant SD of nightly TST
Social jetlag	1.23 ± 1.06 h	0.05 – 4.90	Hours; absolute difference in midsleep between workdays and free days
SRQ Circadian Regularity subscale (sum)	8.64 ± 2.84	0 – 14	Sum of 4 items (0–16); higher scores indicate more regular bed and wake timing
SRQ Sleep Continuity Regularity subscale	5.02 ± 2.09	0 – 8	Sum of 2 items (0–8); higher scores indicate more regular awakenings and time awake at night
SRQ Global score	13.66 ± 4.08	4 – 21	Sum of 6 items (0–24); higher scores indicate more regular perceived sleep timing and continuity overall
